# Demonstrating the reproductive consequences of cognition: learning from experience

**DOI:** 10.1098/rstb.2024.0115

**Published:** 2025-06-26

**Authors:** Susan D. Healy

**Affiliations:** ^1^ University of St Andrews School of Biology, St Andrews, UK

**Keywords:** cognition, experience, fitness, food storing, nest building, reproductive success

## Abstract

Reproductive success and its consequences for fitness are key to determining how natural selection shapes cognitive abilities, and thus how and why cognition might change over evolutionary time. However, demonstrating reproductive costs or benefits owing to variation in cognitive ability is hugely challenging, in part because traits such as body size and condition make the largest contribution to reproductive success. Our ability to discriminate a role for ‘being smart’ in fitness is then already limited. Additionally, most animals have many ways to solve problems, only some of which will depend on their cognitive abilities. However, specific instances in key parts of animal’s life may depend heavily on their cognitive abilities, and without them the animal will not survive or reproduce. Retrieving food stores is one example and nest building might be another. For food-storing, spatial ability is key, while for nest building the pertinent ability is learning what works. The importance of animals learning from previous experience may be very common but is rarely considered explicitly in cognitive terms. Work on fitness consequences of cognition should develop from the presentation of discrete tasks testing specific abilities to encompass how learning from experience influences behavioural evolution more broadly.

This article is part of the Theo Murphy meeting issue ‘Selection shapes diverse animal minds’.

## 1. A bit of history to start

Animal cognition has been an integral part of behavioural ecology since the early days of optimal foraging. It became clear that the addition of learning and memory to foraging models consistently improved the explanatory power of those models (mostly rodents, e.g [1–4]). However, with the advent of a body of work on food storing in songbirds, beginning in the early 1980s [5,6], cognition itself became the focus of interest; specifically, the cognition of wild animals in ecologically relevant contexts rather than of laboratory-tested model species that had been part of comparative psychology for years (e.g. [7–11]). However, even more importantly, these questions were not directed at describing what psychological attributes those animals exhibited or how those abilities did or did not contrast with the similar psychological attributes known for humans. Rather, the questions were raised in a classic behavioural ecology functional (i.e. ultimate) framework, with a cost-benefit analysis to the fore. The nature of hypotheses under test was of the kind: as learning and memory will be costly, animals that are better able to learn and remember X (e.g. spatial locations) must benefit in some way (e.g. [12–16]). That benefit should, ultimately, be one of greater fitness, as better learning and memory capacity will enable the possessor to outcompete its more forgetful conspecifics. In the shorter term, the benefit might be more conspicuous in terms of better foraging ability, or in the case of food storing, a better memory for food stores.

Spatial learning and memory, in the form of food storing, became the system of choice for, as is usual, both logistic and theoretical reasons. By the 1980s quite a lot was known about spatial navigation, both from the laboratory, mostly on rodents (e.g. [17–21]) but also from a thriving body of work on homing in pigeons (e.g. [22–25]). In addition, not all species store food; indeed, rather appealing was the fact that in Europe at least, there were songbirds within the same family that differed in the degree to which they exhibited this behaviour (the Paridae and the Corvidae). How food storing had evolved had become interesting [26] as the number of items stored, the duration before stores were retrieved, and the inconspicuousness of the storage sites, seemed to pose a significant problem for the storer. Because it was challenging to examine the details of memory use for food stores in free-living animals (mostly birds [27,28]), several research groups moved to capturing wild birds and taking them into the laboratory for testing (e.g. [11,29–31]). The work that built on these early studies grew into the broader body of spatial navigation, and into neural analyses, especially of the form and function of the hippocampus, which is the part of the brain known to process spatial information, including that for memory of food stores [32–34].

By the early 2000s, other questions had begun to attract interest in the realm of cognition in wild animals, especially that of behaviours such as tool use (e.g. [35–37]) and problem-solving (e.g. [38,39]). This redirection of attention was coupled with a new generation of behavioural ecologists becoming curious about (and indeed, confident of) the cognitive abilities of their species, with an unprecedented increase in diversity of species and systems appearing in the literature (e.g. various bowerbirds, songbirds and fishes: [40–44]). The greater proportion of tests of problem-solving in wild animals involved presenting them with one or other kind of box containing food (e.g. [41,45–47]), which then allowed testing of a much wider variety of wild animals in the wild than had been achieved in the preceding decades. Technologies such as Radio Frequency Identification Tagging (RFID) and programmed electronic food-dispensers deployed in the field have also played a role in enabling researchers to address questions such as how information spread through groups of individuals (e.g. [48,49]).

## 2. Testing cognition in the wild

There are multiple examples in which cognition in the wild has been examined. A pre-eminent example is that of food storing. Owing to technological advances, an influential body of work has been produced by Pravosudov and collaborators in particular (see [34] for a review of that work). Many of the other examples involve the deployment of boxes containing food rewards for the animal to try to open using one or other methods (e.g. [50–53]). Some of the studies are directed at the transmission of information through a population (e.g. [47,54]), but many look to quantify cognitive performance. The key issue here is ecological salience or relevance. Ideally, we would like to test the cognitive abilities that have evolved in terms of the ecological conditions, biotic and abiotic, in which those abilities evolved (e.g. [55–57]). Because they are interested in how psychological concepts and neural substrates are shared across animals, comparative psychologists are rightly happy to ask how many other species can open a box like a human [58]. Behavioural ecologists, however, ask a different kind of question: why did this particular cognitive ability evolve? To answer that question requires consideration of the various foraging, mating, socializing and avoiding predation tasks an animal faces in real life, and how cognition may or may not increase an individual’s fitness across one or more of those contexts.

**Figure 1.**
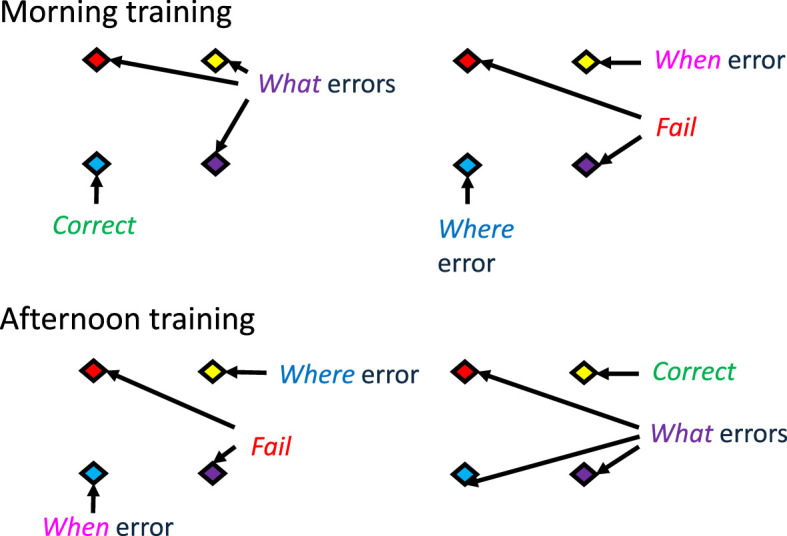
Schematic of the experimental design for testing episodic memory in free-living wild rufous hummingbirds. Birds were presented in their territories with eight artificial flowers, one of which contained a small amount of sucrose. The flowers were presented in two identical arrays of four flowers in the morning and again in the afternoon. In this example, in the morning, the blue flower in the left-hand array contained food, and in the afternoon, the yellow flower in the right-hand array contained food. Visits to the flowers could be categorized as *correct* or into various kinds of errors: *where* errors (the bird visits the correct flower for that time of day, but the flower is in the wrong array), *what* errors (the bird visits the correct array for that time of day, but the colour of the flower is wrong), *when* errors (the bird visits the correct flower for that array but for the wrong time of day), and *fail* (none of the features of the flower are correct [66].

A system where ecological validity has been used to study cognition in the wild is spatial memory and foraging in wild, free-living rufous hummingbirds (*Selasphorus rufus*) in which birds are presented with artificial flowers that contain sucrose (sugar and water; e.g [59,60]; see similar kinds of experiments with bees, for example [61,62]). Naturally occurring flowers may differ in a range of features such as colour, content, location, number and/or the rate at which they are refilled, all of which can be experimentally replicated with artificial flowers in their natural habitat [63,64]. Their spatial learning and memory capacities are such that they learn a flower’s location after a single visit, and a flower’s refill rate after only a handful of hours of experience. Along the way something of their episodic memories ([65,66]; see figure 1 for a schematic of an experimental design), numerical capacities [67,68] and concept learning [64,69] have been uncovered. Because these birds prefer to feed on the experimental flowers over natural flowers, concerns about the impacts of attention and motivation on the birds’ performance, which affects all assessments of cognitive ability [70] are reduced, at least to some degree. However, although there is considerable ecological validity underpinning these experiments, our understanding of the evolution of the demonstrated abilities rests on assumptions made about maximizing energy intake while the bird completes a task rather than measures of reproductive success. Using success at obtaining food rewards as a proxy for fitness is also common to nearly all the other experimental tests of cognition with wild animals.

There are, however, multiple examples of correlations between performance on experimental tasks and *survival*. While data on inclusive fitness are the gold standard for determining the fitness impact of variation in a trait, survival is a much more accessible proxy. Also, although in a handful of cases, such as the common pheasant (*Phasianus colchicus*), where individuals that do *more poorly* on cognitive tasks unexpectedly have better survival [71], improved survival has been correlated with better performance in a variety of experimental tests of cognitive ability. For example, juvenile mountain chickadees (*Poecile gambeli*) that do better on a spatial learning tasks are also more likely to survive to their second year [72], grey mouse lemur individuals (*Microcebus murinus*) that did better on several cognitive tests then lived longer than individuals that did less well [73], and for striped mice (*Rhabdomys pumilio*), individuals that did better on an inhibitory control task were also more likely to survive [74]. These latter two studies are examples of a very common approach to testing cognitive abilities in wild animals, which is to use either one or a battery of tasks that are designated as cognitive tests (e.g. [75–77]) because they have been taken ‘off the shelf’ from experimental psychology paradigms [78]. These tasks are similar to those used in experiments that are commonly labelled as ‘problem-solving’, which may or may not actually be tests of one or other cognitive ability [79,80].

The various shortcomings of these off-the-shelf-tasks for providing compelling evidence for cognitive differences between species or individuals have been discussed for more than 40 years [81] with occasional reminders (e.g. [70,82]), but there always seems to be a reason to keep on with them. One of those reasons is that they seem to have clear, testable outcomes with regard to psychological performance (e.g. tests for inhibitory control: [83,84]). They have been used in the laboratory to test model animal systems for years, and the methodology has been shown to be robust across multiple species and contexts. The problem is that after at least a decade in which tests of this kind have been used in the field, they have not yet led to the promised land: while survival benefits have sometimes been demonstrated (and we should not forget the considerable effort that needs to be exerted to collect these data), we still do not have clear and compelling data that better cognitive performance leads to greater *reproductive success*. While even survival data are challenging to collect, they remain a *proxy* for fitness.

## 3. Age and experience

At least one journal issue has been devoted to the problem of identifying reproductive success with cognitive performance. In that issue [85], multiple ideas and approaches for dealing with this problem were proposed, and there is much that is worthy of consideration in those collected papers. Here, however, I would like to consider a further factor that might provide a way to evaluate the fitness effects of cognitive ability, namely the role of ‘experience’. In terms of the studies of birds, the importance of breeding experience—which has nearly always been coupled together with age—has long been considered for its role in enhancing reproductive success (e.g. [86,87]). More generally though, the role of experience across foraging, mating and social contexts has been studied in many species, vertebrate and invertebrate alike. Here, I focus on birds and estimates of reproductive success.

Age has often been shown to be strongly associated with breeding success in birds (e.g. [88–90] and many later examples). Inexperienced breeders typically do less well than more mature individuals, while breeding success often then declines as individuals age further. Older breeders may also increase their reproductive effort e.g. California gulls *(Larus californicus)* [91] and glaucous-winged gulls (*Larus glaucescens*) [92].

There are a number of reasons why reproductive success may vary as an individual ages, alongside changes in reproductive physiology, all of which could interact with each other. First, selection may favour changing patterns of investment in reproduction across breeding attempts, for instance changing reproductive ‘effort’ across attempts, in terms of clutch size, levels of parental care or attempts to breed again after a failed attempt (e.g. [91,93–95]).

Second, one factor that has received a considerable amount of interest has been the role played by the ‘coordination’ of parental effort between the two birds in a pair [96,97]. This often seems to increase when a pair attempts a second round/year of reproduction, such as in great tits (*Parus major*) in the United Kingdom (e.g. [98,99]). In relatively short-lived birds, the effect of within-pair coordination may be small (e.g. thorn-tailed rayaditos *Aphrastura spinicauda* [100]) or not visible (e.g. mountain chickadees [101]), but positive effects have been commonly observed in long-lived species, such as the glaucous-winged gull (*L. glaucescens*) [92].

Although it has been demonstrated many times, how coordination between mates leads to reproductive benefits remains unclear. The benefits of mate retention might be somewhat straightforward when a mate is lost (depending on when that occurs) as a newly introduced pair may begin their reproductive event later than pairs that are already together and can embark sooner [102,103]. Although some parts of a reproductive event such as building the nest could be sped up, egg laying cannot, except if fewer eggs are laid. On the other hand, it may be that the benefits of coordination are all about how the parents deal with the offspring, such as synchrony in visits to the nest as in long-tailed finches (*Poephila acuticauda*) [104] and zebra finches [105], or in the alternation of parental feeding visits as in the long-tailed tit (*Aegithalos caudatus*) [106], all of which lead to increased reproductive success. That said, coordination in offspring provisioning is not always associated with clear reproductive benefits, for example, house sparrows (*Passer domesticus*) [107].

Third, reproductive experience may drive the correlation between reproductive success and age. Of course, experience is a very broad term, and many aspects of a bird’s breeding habitat and ecology may shape reproductive decisions, but here I am specifically interested in the experience of a successful breeding attempt, and what animals might learn, if learn they do, about or from that successful attempt. For all birds, what happens to their eggs (or the eggs laid by close relatives as in cooperative breeders) and/or chicks that hatch from those eggs is crucial to their reproductive success. For those birds that build a nest into which they lay their eggs, the point of the nest is to enhance the chances that their eggs are incubated successfully, which means protecting them from the external environment (e.g. the weather and predators). It seems plausible that those avian builders that survive long enough to reproduce more than once would have a selective advantage if they used information they acquired from the first experience to modify their building decisions second time around. Mistakes made while foraging such as taking only the largest items or staying too long in a patch, as long as they are not fatal, can probably be corrected without being too costly. Not producing live babies from a reproductive event, especially if the opportunity to reproduce is seasonal/annual, may mean that the adult must survive many months before another opportunity arises. When it does get that opportunity, if it can do anything to improve on its last effort, we might expect them to try to do so. There is a plenty of scope here for a behavioural ecologist interested in the role of cognition in the decision-making of their birds during reproduction: mate choice and rearing of the offspring have long drawn the focus of effort, but the step in-between might merit some attention.

There are various factors concerning a reproductive event that a bird might learn or associate with that experience. For example, if eggs are laid but do not hatch, the builder, male or female, may associate the choice or amount of material used to build the nest with the outcome and decide that the previous choice had been inappropriate or insufficient (as seems to be the case in laboratory zebra finches, see next section), and therefore make different choices when building its next nest. On the other hand, if eggs or chicks are lost to predators, the nest builder might consider that the choice of nest location had been an error that could be corrected, by moving to build the next nest in a different location. Similarly, if the nest blows down, the chicks die of heat exposure, or owing to some other climatic infelicity, the builder with or without their partner might choose to build in a different location, but one that specifically addresses the cause of chick death. Alternatively, if chicks grow very slowly or die of starvation, perhaps the problem is the mate or the helpers at the nest, and something about that partnership needs work before embarking on a subsequent breeding attempt. If, however, a breeder is successful in producing fledglings, they might be expected to change as little as possible.

Indeed, many birds vary in whether they move breeding site between reproductive attempts. Furthermore, birds often move after being reproductively unsuccessful, with American robins (*Turdus migratorius*) and brown thrashers (*Toxostoma rufum*) being just two examples of species that do this [108]. Confirmation that this is a decision rather than mortality-induced disappearance, of course, requires data on birds at the new sites, not just the disappearance from a site following a failed breeding attempt. Long-term datasets offer evidence of such movement decisions. For example, 40 years of data on breeding tawny owls (*Strix aluco*) has shown that owls which lose a mate tend to move territory, in search of a replacement partner [109]. Alternatively, birds may have to learn how to care for young, as seems to explain the increased foraging effectiveness of helpers at the nest in brown jays (*Cyanocorax morio*) as they aged [110] and in superb fairy-wrens (*Malurus cyaneus*) [111].

Again, long-term datasets, especially from longer lived birds, might be a source of evidence for (or against) such effects on decisions. In lance-tailed manakins (*Chiroxiphia lanceolata*), for example, females use their experience of reproductive success to choose whether to change the location of their subsequent nest, even though that choice does not necessarily lead to them to success in that next nest. Successful females were more likely to stay to nest again in a site than were unsuccessful females. Age of the females did not explain the pattern of decision-making and although this lance-tailed manakin data provide support for the win-stay-lose-shift hypothesis as an explanation for moving nest site (as there has been for a range of other bird species), success in the most recent previous nest was the best explanation of current success. Taken together, these data suggest that manakins have taken something from their previous experience and applied it to their current situation. In this situation, as in many of the examples in which reproductive experience seems to affect subsequent decision making, exactly what the bird has learned and how it uses that knowledge for its next breeding attempt is unclear. Perhaps it has learned better how to forage and feed its offspring, as above, it has learned how to coordinate feeding with its partner, how better to reduce predation risk—there are multiple possibilities.

## 4. Learning, experience, and nest building

As suggested above, an additional but probably very important contributor to reproductive success in birds and many other species is the nest in which the young are incubated and initially reared. Also, there is emerging evidence that builders do, indeed, come to associate features of nest site choice or even features of the nest they build, with their own reproductive success. To begin with, there is now clear evidence that learning can be involved in a variety of components of nest building, including learning how to handle material (e.g. southern masked weavers (*Ploceus velatus*) [112]), learning what material to use from just looking at a nest (e.g. zebra finches (*Taenyiopygia guttata*) [113]) and learning to avoid building with costly materials [114]. This latter result also has a putative explanation as zebra finches that used more pieces of the costly material to build their nest produced fewer fledglings [115].

**Figure 2.**
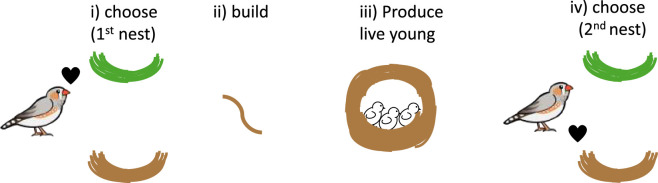
Schematic of an experiment in which zebra finches were asked to choose between nest building material that was either green or brown: i). They then were given either their preferred or their non-preferred colour of material to build their first nest: ii), and then they did or did not go on to be reproductively successful (experimentally manipulated): iii). If the birds produced live young and built with material of the colour they did not prefer, they switched their preference of material for their second nest, to that initially non-preferred colour: iv) [116].

At least some of these reproductive consequences of nest building affect subsequent building decisions. For example, zebra finches made to build with material of a colour they initially did not prefer, subsequently preferred that material if the first nest successfully produced live young ([116]; figure 2). In this case, for their next nest successful birds *changed* their preference to stick with the material that had worked for them as did successful birds that had built with material of their preferred colour stuck with their preferred colour. These birds, then, both associated material colour with reproductive success.

**Figure 3.**
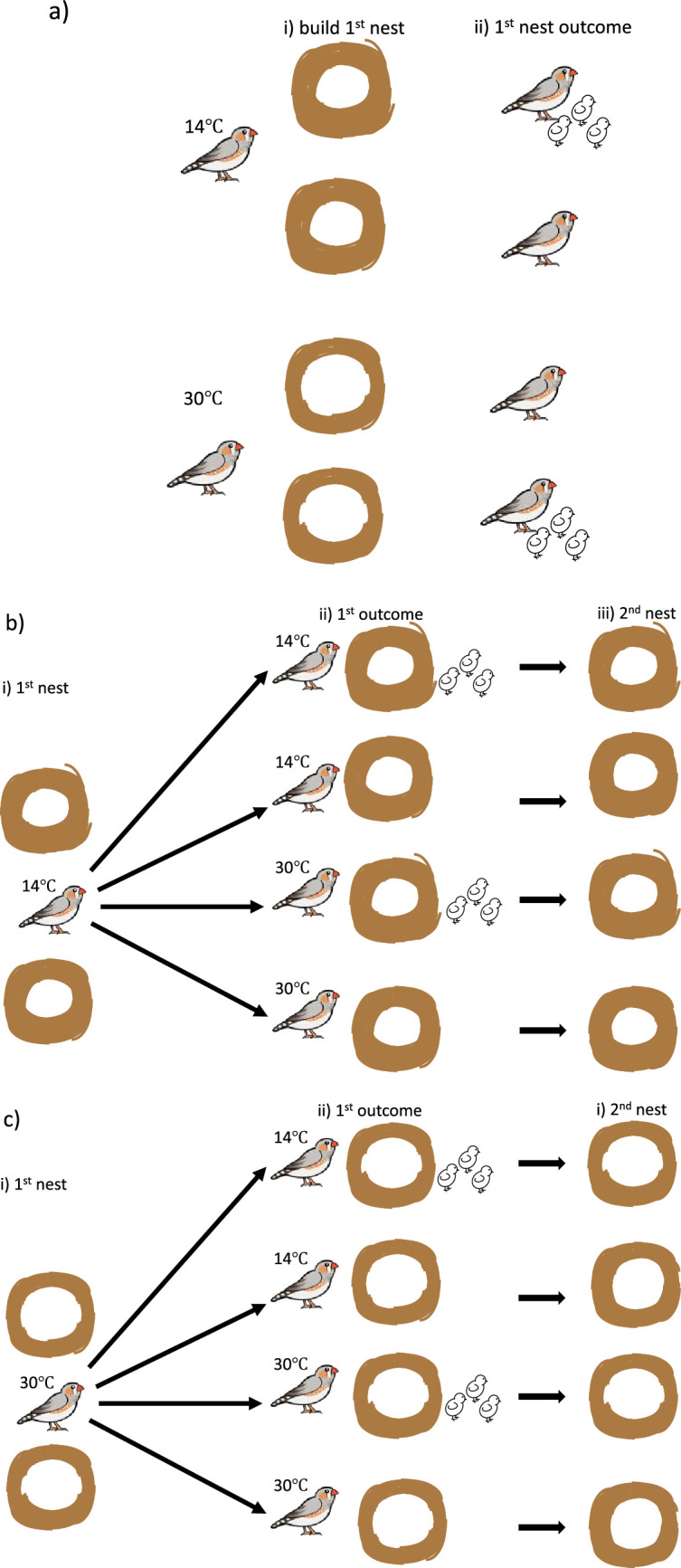
(a) Schematic of an experiment in which zebra finches built their first nest either in a room held at 14°C or at 30°C. i) Birds that built at the cooler temperature put more pieces of material into their nest. ii) Birds that put more material into their nest at either temperature were more likely to produce live young. (b) For their second nest, ii) birds that had built at 14°C were housed at either 14°C or 30°C. iii) Irrespective of the room temperature, birds that had been successful in producing live young from their first nest put the same number of pieces of material into their second nest. Those birds that had been unsuccessful with their first nest put more pieces of material into their second nest. (c) For their second nest, ii) birds that had built at 30°C were housed at either 14°C or 30°C. iii) Irrespective of the room temperature, birds that had been successful in producing live young from their first nest put the same number of pieces of material into their second nest. Those birds that had been unsuccessful with their first nest put more pieces of material into their second nest [117].

Similarly, when building their first nest in experimental rooms set at either 14°C versus 30°C, zebra finches that built their nest in the room that was set at the lower temperature used more material ([117]; figure 3a–c). Birds that included more material into their nests had warmer nests and were more likely to produce live young. Following a switch of some birds to the room of the alternative temperature, some birds built their second nest in a room at the same temperature as their first, while other birds built their second nest at the alternative temperature. For the second nest, the room temperature no longer explained the amount of material with which birds built their nest. Instead, they used an amount of material that was explained by their previous reproductive success, irrespective of room temperature. Successful birds in this experiment again *stuck with* their material use decisions, as did successful builders in an experiment in which they were offered familiar or novel materials for their second nest [118]. In both experiments, for their second nest, the unsuccessful birds either changed their material choices or behaved as if they tried to do something different from their building of their first nest. While the most convincing data thus far comes from laboratory experiments with zebra finches, there also seems to be some evidence that birds in the wild also respond to their own experience of reproduction when they come to build their next nest: successful blue tits (*Parus caeruleus*) add insulation to their nests sooner than do unsuccessful birds and then do so in a similar fashion for their next nest [119]. Unsuccessful birds vary what they do however, with some adding insulation sooner than they had for their first nest, which then leads them to be more likely to successfully fledge offspring. Moreover, unsuccessful individuals are also more likely to change their mate and to move nest box for their next building attempt than are successful birds [120, in preparation].

One problem with working on nest building is the time and effort required to collect substantive quantitative data, especially if like blue tits, the birds take multiple weeks to build their nest. Data on building often means video recording, and many of us know the significant downside to collecting lots of video data: even more time spent analysing them. One alternative option might be to look at just the nest itself. A lot has already been usefully learned from examining nests at the end of the breeding season about the materials the builders used and whether those materials were likely to have helped the parent safeguard their offspring (e.g. [121–125]). Some of those data come from deconstructions of the nest to determine the materials used, while others have come from nests weighed once the young (if there have been any) have fledged (e.g. [126,127]) or from measurements of nest height (e.g. [128–130]). Photographs of the nest taken across the days or weeks of building can be used to examine rate of building [119].

In some circumstances, the impact of experience of building has been observed from measurements of the nest itself [131]. In zebra finches building in the laboratory, , birds that remain together as a pair build a nest of a more similar morphology (using geometric measurements and entrance orientation) than do birds that have different mates for each nest. In that experiment, birds were not allowed to lay eggs or raise chicks so the question of the relationship between reproductive experience and building morphology is still to be addressed. In other examples, however, such as data from mountain chickadees [130,132], it appears that nest builders do not use experience of their previous reproductive outcome to change the size of the nest they build. There are so few species that contribute data to this question at this stage, however, that it does not seem sensible to make sweeping statements or even predictions as to whether we might expect builders to learn from their experience or not. This especially seems to be the case when two closely related species, blue tits and mountain chickadees, appear to differ in whether they use reproductive experience when making building decisions. However, one can certainly ask the question!

It is also possible that taking a single measure of nest structure as for nests of the mountain chickadees (average nest height), rather than looking at the series of decisions about building that occur over the course of days or weeks, might miss important decisions. For example, blue tit builders remove material on around 20% of their building visits and up to 40% of the weight of the nest when measured after the young fledge is not owing to material brought by the builder [133]. Furthermore, insulative capacity of a nest may be inversely related to the success of that nest: for blue tits, a nest that has produced live fledglings has much less insulative capacity than does one from which no young were produced [134]: nests from which live fledglings have been produced are flat and compacted owing to the young growing, moving and defecating in the nest, while the nests from which no young have been produced are much more pristine. Deconstructing nests into their composite materials can also provide useful insight into the different materials the builder has used and which may contribute in different ways to a nest. Blue tits, for example, use moss and grasses for much of the bulk of their nest, while insulatory materials such as feathers and animal hair occupy a much smaller proportion of the nest but play a bigger role in creating ideal incubation conditions. It is these insulation materials that experienced blue tits change most with their subsequent nests [119].

Nest deconstructions are also showing the increasing inclusion of anthropogenic materials (including plastics) into nests (e.g. [135–138]). While this is often raises concerns about the health of the builders and their young, the inclusion of such materials do not always seem to be detrimental, and on occasion may even be beneficial, as for Chinese bulbuls (*Pycnonotus sinensis*) that had greater hatching success the more plastic they had included in their nest [139]. One question that might be asked is whether these birds added just as much, or more plastic, to their subsequent nest. As birds add more and more anthropogenic materials to their nests, it seems one might ask whether they base this choice on their own knowledge of the material’s structural or functional attributes (knowledge that could be experience-dependent) and whether builders can learn about anthropogenic materials so that they choose/avoid them for building subsequent nests as do zebra finches faced with building with stiff or flexible string [114]. Might, for example, seabirds that lose young to entanglement with fish netting or similar, avoid those materials next time? It does seem that house finches (*Carpodacus mexicanus*) both benefit from including cigarette butts into their nests (greater hatching and fledging success: [140]) and decide the amount of cigarette butt fibre to include in their nest based not just on current but also on past parasite loads [141].

## 5. Conclusion

The nest building data show that some birds can associate the outcome of their reproductive attempt (e.g. production of live fledgings) with their nest building decisions. This is an example of the importance that cognition can and does play in the lives of animals and one that has been little explored to date. There are a multiplicity of species and questions that can be addressed, some of which are included above, and a variety of methods that range in their ease and expense of implementation that have already been shown to be useful. Also of course, nest building is not limited to just birds: there is a wide diversity of species that build, providing a wealth of further possibilities.

One need not be confined to bird nest building, however, as the behavioural ecology literature from oviposition-site choosing insects, socially interacting primates or foraging reptiles, is rich in examples in which learned experience benefits fitness [65]. Taking a broader scope may allow us increasingly to understand the extent to which natural selection shapes cognition. To do this, it would help to clarify the role of learning and memory in instances of ‘experience’ and not just take it for granted, to measure experimentally both the benefits *and* the costs of an animal acting on its experience, and then to partition out the selective benefits of experience from the many other components of fitness that shape animal populations. This is likely not only to bring new insights into the shaping of animal cognition by natural selection but also provide a new context for looking at individual differences in cognition, for those who follow or turn their backs on experience [142].

## Data Availability

This article has no additional data.
